# Interference lithographic nanopatterning of plant and bacterial light-harvesting complexes on gold substrates

**DOI:** 10.1098/rsfs.2015.0005

**Published:** 2015-08-06

**Authors:** Samson Patole, Cvetelin Vasilev, Osama El-Zubir, Lin Wang, Matthew P. Johnson, Ashley J. Cadby, Graham J. Leggett, C. Neil Hunter

**Affiliations:** 1 Department of Chemistry, University of Sheffield, Brook Hill, Sheffield S3 7HF, UK; 2 Department of Molecular Biology and Biotechnology, University of Sheffield, Western Bank, Sheffield S10 2TN, UK; 3 Department of Physics and Astronomy, University of Sheffield, Hicks Building, Hounsfield Road, Sheffield S3 7RH, UK

**Keywords:** nanofabrication, photosynthesis, self-assembled monolayers, interferometric lithography, antenna, light-harvesting complex

## Abstract

We describe a facile approach for nanopatterning of photosynthetic light-harvesting complexes over macroscopic areas, and use optical spectroscopy to demonstrate retention of native properties by both site-specifically and non-specifically attached photosynthetic membrane proteins. A Lloyd's mirror dual-beam interferometer was used to expose self-assembled monolayers of amine-terminated alkylthiolates on gold to laser irradiation. Following exposure, photo-oxidized adsorbates were replaced by oligo(ethylene glycol) terminated thiols, and the remaining intact amine-functionalized regions were used for attachment of the major light-harvesting chlorophyll–protein complex from plants, LHCII. These amine patterns could be derivatized with nitrilotriacetic acid (NTA), so that polyhistidine-tagged bacteriochlorophyll–protein complexes from phototrophic bacteria could be attached with a defined surface orientation. By varying parameters such as the angle between the interfering beams and the laser irradiation dose, it was possible to vary the period and widths of NTA and amine-functionalized lines on the surfaces; periods varied from 1200 to 240 nm and linewidths as small as 60 nm (*λ*/4) were achieved. This level of control over the surface chemistry was reflected in the surface topology of the protein nanostructures imaged by atomic force microscopy; fluorescence imaging and spectral measurements demonstrated that the surface-attached proteins had retained their native functionality.

## 1. Introduction

Diagnostic devices [1,2], biomaterials, tissue engineering [3,4], proteomics [5], medical diagnostics [6] and many other applications require the controlled attachment of interacting biomolecules to solid substrates. In particular, the fabrication of bioinspired photovoltaic devices, which ultimately requires the directed immobilization of chlorophyll–protein complexes onto a variety of substrates, is of special interest [7–13]. The well-characterized and relatively simple photosynthetic apparatus of purple phototrophic bacteria is an ideal starting point for such work. The light-absorbers in these organisms are bacteriochlorophyll and carotenoid pigments that bind non-covalently to transmembrane polypeptides, forming ring-like antenna structures. Energy absorbed by arrays of light-harvesting LH2 complexes migrates to the LH1 complex, which surrounds the reaction centre complex where absorbed energy is transiently stored in a series of electron transfer reactions [14–16]. In plants, the LHCII complex absorbs solar energy, which migrates to the photosystem II reaction centre [17–21].

Several studies report the attachment of these and other photosynthetic complexes to various surfaces [7–13,22–25]. A number of techniques such as dip-pen nanolithography [26–31], scanning near-field lithography [32–35], electron beam lithography [36,37] and nanoimprinting [38–40] can achieve nanopatterning. Previous studies using the LH2 complex from the photosynthetic bacterium *Rhodobacter* (*Rba.*) *sphaeroides* and the LHCII complex from spinach have already shown that scanning near-field photolithography [41] and nanoimprinting [5,42,43] can direct the nanoscale surface patterning of these complexes, and that 80-nm-wide lines of LH2 exhibit long-range (micrometre scale) energy transport [44]. However, the ability to easily change the size and the period of nanostructures, and to fabricate them over very large (cm^2^ and larger) areas, still presents a challenge. Here, interferometric lithography (IL) [45] offers a very promising, low-cost, reliable and scalable technology for fabricating nanoscale periodic patterns over large (cm^2^) areas.

In this work, we used IL to photopattern self-assembled monolayers (SAMs) deposited on gold substrates [46,47]. Exposure of amine-terminated SAMs in a dual-beam interferometer led to periodic photo-oxidation of thiolate head groups, enabling replacement of oxidized molecules by contrasting oligo(ethylene glycol) functionalized adsorbates. Derivatization of intact amine-terminated adsorbates either with an imido-ester cross-linker or with nitrilotriacetic acid (NTA) enabled the specific immobilization of either native or polyhistidine-tagged light-harvesting complexes. Proteins were bound specifically to the Ni^2+^–NTA regions with very little non-specific adsorption. The periodicity of the interference pattern was controlled by varying the angle between the two interfering beams, and the width of the modified regions of the SAM was broadened by increasing the exposure to the laser irradiation. We show that polyhistidine (His)-tagged RC-LH1-PufX complexes from *Rba. sphaeroides* and LHCII complexes from spinach can be precisely immobilized to form parallel lines of predetermined widths and periodicities. *In situ* fluorescence emission spectroscopy confirmed the retained functionality of these nanopatterned complexes.

## 2. Material and methods

### 2.1. Chemicals

11-Amino-undecanethiol hydrochloride (99%), triethyleneglycol mono-11-mercaptoundecyl ether (95%; OEG-thiol) and 25% glutaraldehyde solution were purchased from Sigma Aldrich. Ethanol (HPLC grade) was purchased from Fisher Scientific Limited (Loughborough, UK). *N*-(5-amino-1-carboxypentyl) iminodiacetic acid (AB-NTA) was purchased from DoJindo Molecular Technologies. All aqueous solutions used in the preparation of NTA surfaces for protein attachment were prepared using ultrapure water (Elga LabWater Systems).

### 2.2. Patterning of His-tagged and native proteins by interferometric lithography

**Figure 1.**
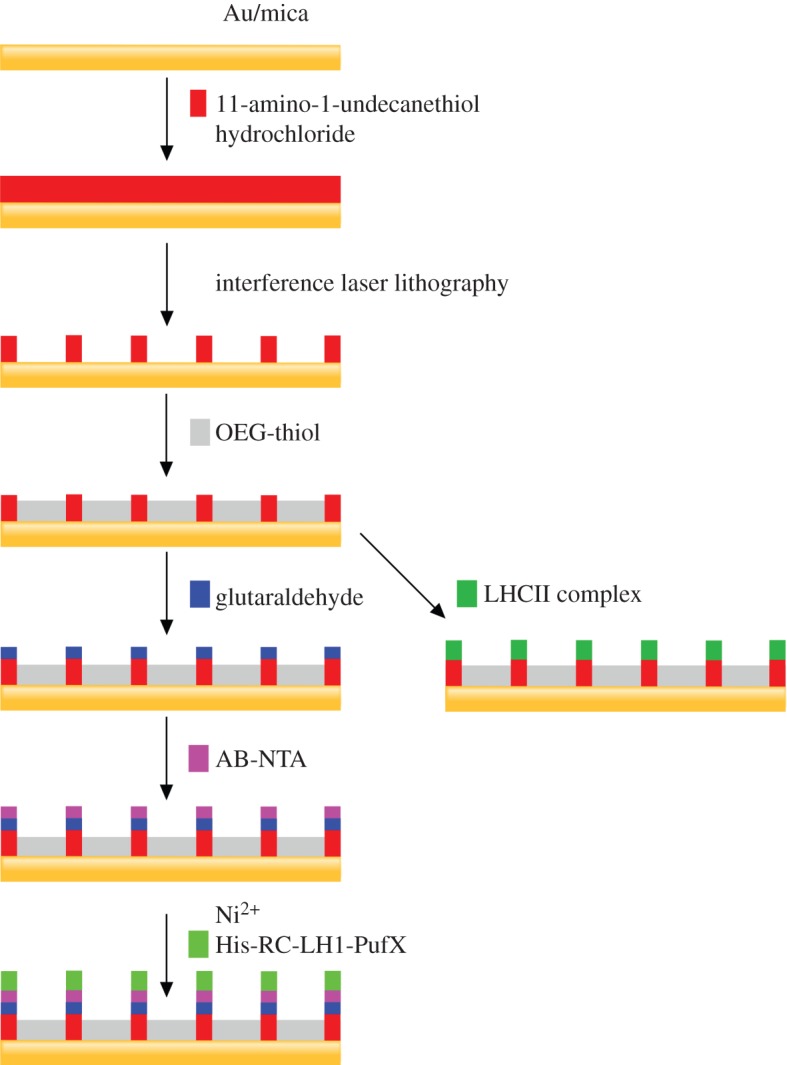
Schematic diagram illustrates the sequence of steps used for immobilizing light-harvesting proteins on gold substrates.

**Figure 2.**
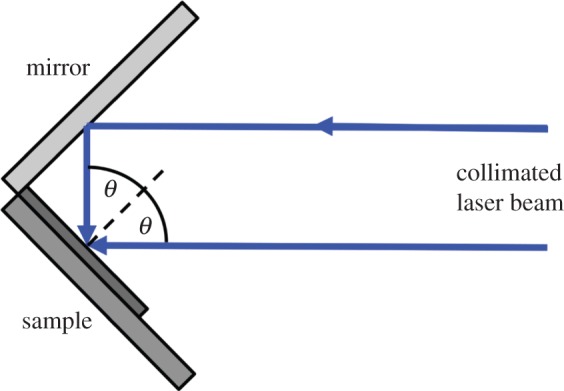
Schematic diagram of the Lloyd's mirror configurations used for IL. *θ* is the half-angle between the two interfering beams.

The scheme in figure 1 illustrates the steps involved in the preparation of IL-patterned lines of NTA for immobilizing His-tagged RC-LH1-PufX and native LHCII proteins. SAMs of amine-functionalized thiols on either 200-nm-thick epitaxial gold layer (Phasis Sárl, Switzerland) or 30-nm-thick polycrystalline gold were formed by immersing these substrates in a dilute (2 mM) ethanolic solution of 11-amino-1-undecanethiol hydrochloride for 18 h at room temperature. After the reaction, the surfaces were rinsed with pure ethanol and blown dry with a nitrogen stream prior to IL photopatterning, which was carried out using a Coherent Innova 300C FreD frequency-doubled argon ion laser (Coherent UK, Ely, UK) emitting at 244 nm with a maximum power of 100 mW. An expanded collimated laser beam was directed onto a Lloyd's mirror configuration [46,48] (figure 2) with a 90° sample and mirror geometry such that half of the beam directly irradiated the sample. The other half of the beam was incident onto the mirror and was reflected back onto the sample. Interference between the two halves of the beam produces an interference pattern of lines with a period *d*2.1
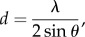
where *λ* is the wavelength of the incident laser beam and *θ* is the half-angle between the two interfering half-beams.

In order to vary the period of the linear nanopatterns, *θ* was adjusted to 6°, 13°, 18° or 30°, and a series of amine-functionalized surfaces was exposed to a laser beam for 10 min at a power density of 64 J cm^−2^.

In order to vary the linewidth of functionalized linear nanopatterns, surfaces with functionalized monolayers were exposed to laser irradiation for various lengths of time ranging from 2 to 15 min (corresponding to an irradiation dose of 14–105 J cm^−2^) at *θ* values of 6°, 13°, 18°, 30°. After the photo-oxidation step, the exposed regions of the gold surface were re-functionalized by immersing the surfaces in a dilute (2 mM) ethanolic solution of OEG-thiol for 10 min, then washed with fresh ethanol and dried with a stream of nitrogen.

In order to create NTA functionalities at the surfaces, patterned surfaces were immersed in an aqueous solution of 200–300 mM glutaraldehyde for 20 min, then washed extensively and repeatedly (five or six times) with a spray of ultrapure water and ethanol. The surfaces were then dipped in an aqueous solution of AB-NTA (pH = 5.2) for 2 h and washed with ultrapure water. Finally, surfaces were immersed in an aqueous solution of 10 mM NiSO_4_ for 8–10 min and then washed extensively with water and blown dry with a stream of nitrogen.

#### 2.2.1. Amine surfaces

The procedure outlined in figure 1 was used, but, after photopatterning, the surfaces were not treated with glutaraldehyde and AB-NTA; instead, the samples were immersed in a 2 mM ethanolic solution of OEG-thiol to produce a protein-resistant layer on the light-exposed regions. These patterned samples were then soaked in phosphate-buffered saline (PBS) for 30 min prior to protein adsorption.

#### 2.2.2. Protein immobilization

NTA-functionalized nanopatterned substrates were incubated with a dilute solution of the His-tagged RC-LH1-PufX complex at a final concentration of approximately 75 nM in imaging buffer (20 mM HEPES, pH 8.3, with 0.03% *n*-dodecyl-beta-maltoside (β-DDM)) for 5–7 min, then extensively rinsed in imaging buffer. For the immobilization of LHCII complexes, the amine-functionalized substrates were treated with 15–20 mM dimethyl suberimidate (DMS) solubilized in buffer containing 20 mM HEPES, pH 8.3, for 30–40 min. After extensive rinsing, the substrates were incubated with LHCII complexes diluted in buffer containing 20 mM HEPES, pH 8.3, and 0.03% β-DDM for 5–7 min at a final protein concentration of 50–100 nM. Samples were stored in imaging buffer at 4°C for atomic force microscopy (AFM) and fluorescence imaging.

### 2.3. Atomic force microscopy

A MultiMode 8 atomic force microscope (Bruker) was used for imaging SAMs and the topology of patterned proteins. Lateral force microscopy (LFM) imaging of photopatterned SAMs was conducted in air at ambient conditions using triangular SNL probes (Bruker) with a nominal spring constant of approximately 0.12 Nm^−1^ and a nominal resonant frequency of around 23 kHz. Immobilized nanopatterned RC-LH1-PufX and LHCII complexes were imaged in PeakForceTapping mode at nearly physiological conditions in buffer (PBS, pH 7.4), at room temperature using SNL probes (Bruker) with a nominal spring constant of approximately 0.35 Nm^−1^ and a nominal resonant frequency of around 18 kHz (in liquid). The modulation amplitude and frequency were adjusted to values in the range 20–24 nm and 2 kHz, respectively.

### 2.4. Fluorescence imaging and spectral measurements

After AFM characterization, patterned samples with RC-LH1-PufX or LHCII proteins were mounted between a microscope slide and coverslip in imaging buffer and were sealed with DPX Mountant (Sigma-Aldrich) prior to fluorescence measurements. The fluorescence emission properties of the immobilized patterned proteins were measured using a home-built inverted epifluorescence microscope (Zeiss Axio Observer.A1m) equipped with a spectrometer (Princeton Instrument Acton 150) and an electron-multiplying charge-coupled device (EMCCD) camera (Princeton Instrument ProEM 512). Excitation was from a collimated light-emitting diode (LED) light source emitting at 470 nm (ThorlabsM470L2), and the resulting fluorescence emission was detected through the spectrometer onto the EMCCD camera.

During the fluorescence imaging and spectral measurements excitation, light was filtered by a 470/40 nm bandpass filter, then reflected by a 605 nm dichroic beamsplitter (SemrockFF605-Di02) onto the sample. For RC-LH1-PufX samples, the fluorescence emission was collected using a 680 nm longpass emission filter (Chroma, Q680lp), and a 593 nm longpass filter (SemrockFF01–593/LP) was used for LHCII. The spectra were captured with a slit width of 800 µm and a 150 g mm^−1^ grating at a central wavelength of either 900 nm for RC-LH1-PufX or 680 nm for LHCII. Each fluorescence image and emission spectrum was an average of 10 frames with a 0.1 s exposure time.

## 3. Results and discussion

### 3.1. Interference periodic patterning of nitrilotriacetic acid surfaces: control of period and line width

Periodic patterned lines of NTA across Au substrates were prepared as described in the Materials and methods. Upon exposure to UV radiation, SAMs of amine-thiols on gold were photo-oxidized [24]. Exposure of alkylthiolate SAMs to UV light in the presence of air causes photo-oxidation of the head-group to yield an alkylsulfonate [49], which can be removed by washing in polar solvents such as water or ethanol; alternatively, alkylthiolate SAMs can be replaced by another thiol, in this case with a OEG-thiol linker. Following photopatterning, the amine-thiols in the unexposed regions were then converted to NTA by reaction with glutaraldehyde and then treated with AB-NTA as described in the Materials and methods, and depicted in figure 1.

**Figure 3.**
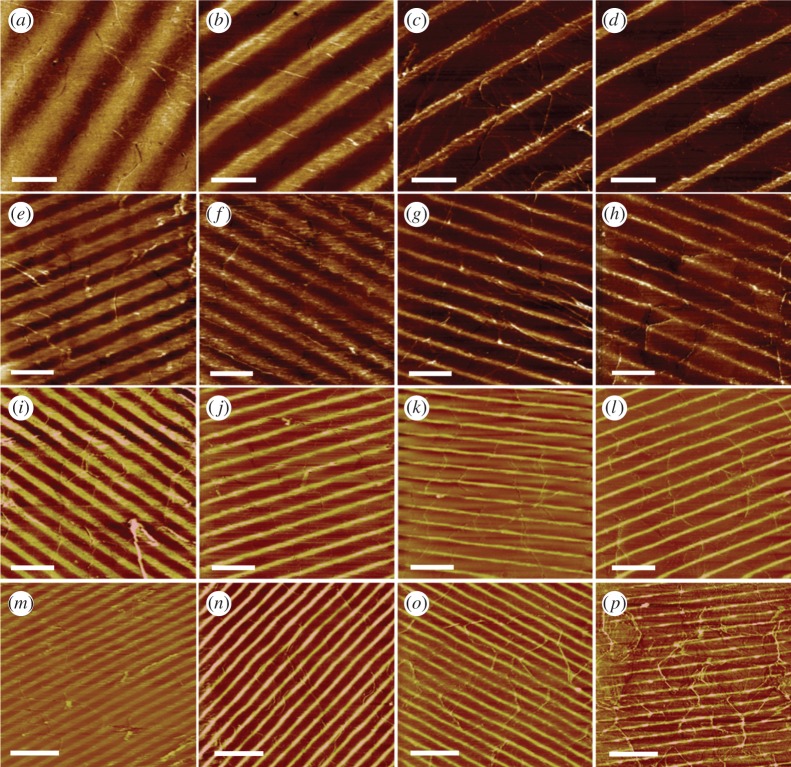
Lateral force microscopy images (5 × 5 µm^2^) showing control over the period and width of lines of NTA on gold/mica achieved by varying the exposure time (typically 2–15 min and with a corresponding dose of 14–105 J cm^2^) at fixed angles of 6° (*a*–*d*), 13° (*e*–*h*), 18° (*i*–*l*) and 30° (*m*–*p*). These data are summarized in table 1. All scale bars, 1 μm.

The LFM images in figure 3*a–p* show periodic patterns of NTA lines obtained with half-angles *θ* of 6°, 13°, 18° and 30°, respectively, between the sample surface and the incident laser beam, and with varying exposures. Alternating bands of bright and dark contrast were observed in every case, with the bright bands arising from the strongly polar NTA groups that adhere strongly to the probe yielding a higher rate of energy dissipation than the less polar OEG-rich regions. The widths of the bright bands decreased with exposure. At lower exposures, the intensity is only sufficient to cause complete photo-oxidation of adsorbates near to the maxima in the interferogram. Consequently, after these narrow bands are derivatized with OEG-thiol, broad bands occupied by amine-terminated thiols remain between them, which yield bright contrast in the LFM images after functionalization with NTA. As the exposure increases, the widths of the bands of photo-oxidized adsorbates increase, thinning the residual amine-functionalized regions to which NTA is attached. This ‘photochemical whittling’ [46] of the unexposed amine regions widens the OEG-thiol regions that are formed after the final adsorption step, and narrows the lines of the remaining intact amine-terminated adsorbates, as shown in table 1.

**Table 1.** Variation in periodicity of lines with the change of half-angle between the two interfering beams, and the variation in line width for different exposure times. The values of periods and widths were measured as the full width at half maximum (FWHM).

half-beam interference angle (*θ*)	period of lines (nm)	width of line (nm)			
		2 min exposure	5 min exposure	10 min exposure	15 min exposure
6	1200	600	400	200	170
13	550	290	210	110	80
18	400	200	100	70	60
30	240	130	100	80	50

### 3.2. Nanopatterning photosynthetic membrane protein complexes onto Ni^2+^–nitrilotriacetic acid lines created by interferometric lithography

**Figure 4.**
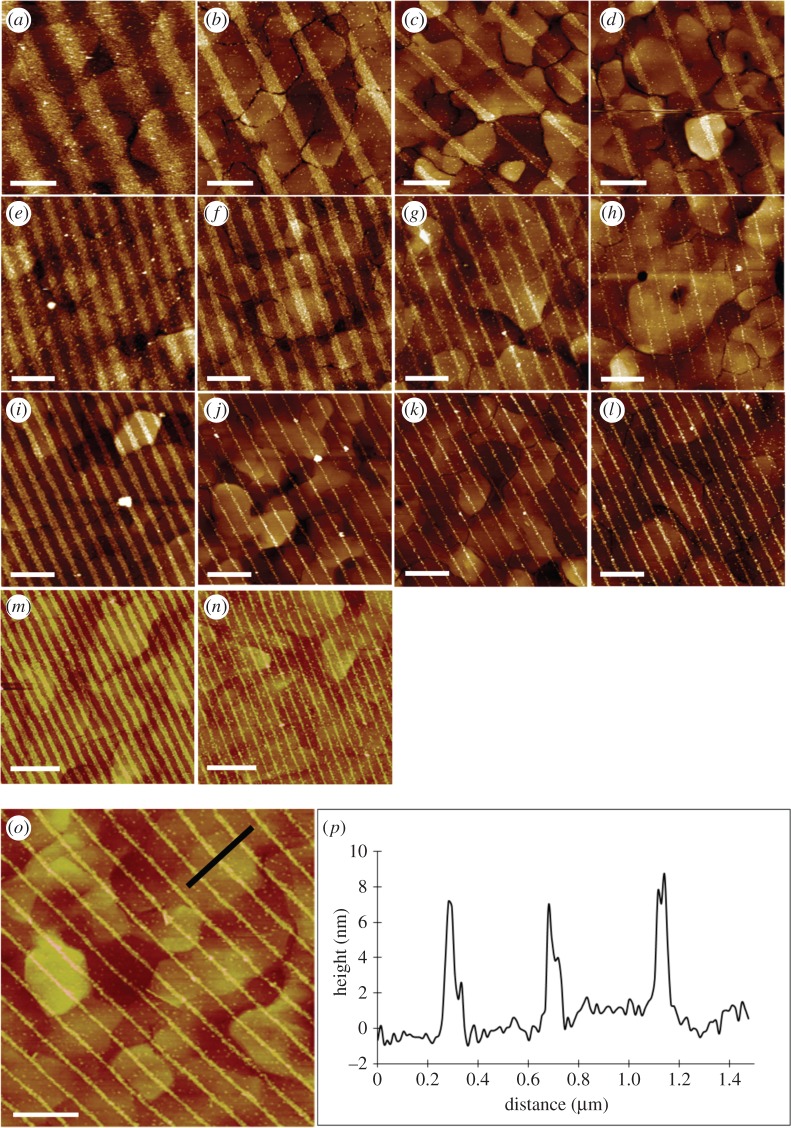
(*a–n*) AFM topographs (5 × 5 µm^2^) of surface-immobilized His-tagged RC-LH1-PufX complexes attached to Ni^2+^–NTA lines produced by IL. The half-interference angles are 6° (*a*–*d*), 13° (*e*–*h*), 18° (*i*–*l*) and 30° (*m*,*n*). For the images running horizontally, the exposure time at a given angle was progressively increased: 2 min (*a*–*m*), 5 min (*b*–*n*), 10 min (*c*–*k*), 15 min (*d*–*l*). (*o*) A section (black line) was taken across three lines of RC-LH1-PufX complexes. (*p*) Height profile corresponding to this section. All scale bars, 1 µm.

The versatility of the IL approach for yielding the variety of NTA lines in figure 3 was exploited for nanopatterning photosynthetic membrane protein complexes. These complexes form two-dimensional arrays in the lipid bilayer membranes of photosynthetic organisms, and their function is to absorb, transmit and eventually store solar energy. For use in our nanopatterning experiments, these complexes are removed from their native lipid bilayer by gentle detergent treatment and purified by several rounds of chromatography. Initially, we used the monomeric RC-LH1-PufX complex from the purple phototrophic bacterium *Rba. sphaeroides*; a medium-resolution structure of the RC-LH1-PufX dimer was published recently [50]. NTA lines were complexed with Ni^2+^ ions then used for attachment of RC-LH1-PufX monomers, genetically engineered to bear a His_10_ tag on the cytoplasmically facing RC-H subunit. This combination of an engineered His-tag and NTA groups on the surface ensures a uniform orientation of the immobilized complexes. The tapping mode AFM topographs in figure 4 (top panels) show that RC-LH1-PufX complexes are attached to the NTA lines, with very little non-specific adsorption and with periods very similar to their parental LFM patterns in figure 3. The 7–8 nm heights of these features, measured across the black line in figure 4 (bottom panels) and displayed as the cross section in figure 4*b*, are slightly lower than measured for two-dimensional crystals of a similar RC-LH1 complex [51], which we attribute to the AFM probe causing small displacement of surface-bound complexes. It is also possible that these complexes adsorb to the surface at a variety of angles, whereas they are held at a fixed position in the two-dimensional crystals.

### 3.3. Assessment of the functional integrity of surface-bound RC-LH1-PufX complexes

**Figure 5.**
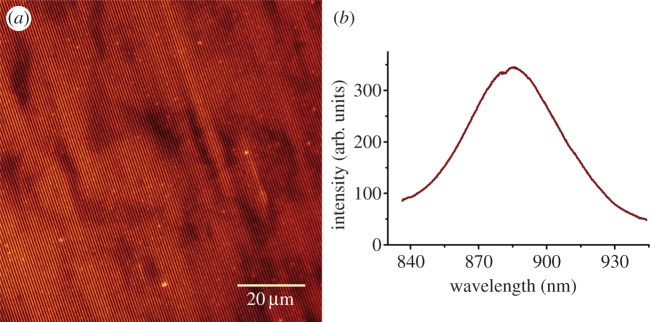
Spectroscopic characterization of RC-LH1-PufX complexes immobilized on Ni^2+^–NTA lines patterned by IL on gold substrates. (*a*) False colour fluorescence image. (*b*) The fluorescence emission spectrum recorded on immobilized complexes.

The fluorescence emitted from the bacteriochlorophyll or chlorophyll pigments of photosynthetic complexes is a useful indicator of their structural integrity and function. In the case of RC-LH1-PufX complexes, a closely packed ring of 28 bacteriochlorophylls, attached to LH1 polypeptides, forms a belt round each RC, the site of charge separation [50]. Monomeric bacteriochlorophylls in solvent emit fluorescence at approximately 780 nm, whereas their assembly into ring-like structures red-shifts their emission by over 100 nm [52]. The presence of red-shifted fluorescence is therefore a useful measure of retained structure and function following immobilization onto IL-patterned Ni^2+^–NTA. To investigate the possible effects of immobilizing RC-LH1-PufX complexes on patterned surfaces, we recorded the florescence emission of Ni^2+^–NTA patterns using a home-built inverted epifluorescence microscope. Patterned core complexes were excited at 470 nm with a collimated LED light source, and the resulting fluorescence was collected through a spectrometer onto an EMCCD camera. The image in figure 5*a* shows the lines of fluorescent RC-LH1-PufX complexes; a region of interest on the pattern was defined by closing the slits of the spectrometer and, using a suitable grating and centre wavelength, the fluorescence emission spectrum was measured (figure 5*b*). The 885 nm fluorescence emission maximum shows that the RC-LH1-PufX complexes have retained their native properties following surface immobilization.

### 3.4. Interference patterning of the plant light-harvesting LHCII complex

In order to explore the possibilities of attaching different types of light-harvesting complexes, we immobilized LHCII complexes purified from spinach thylakoid membranes by using an imido-ester cross-linker on photopatterned amine monolayers on gold surfaces.

**Figure 6.**
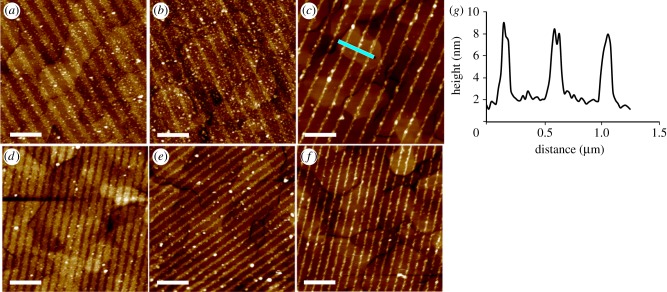
(*a–f*) AFM topographs (5 × 5 µm^2^) of LHCII complexes attached to IL-patterned amine lines. The various LHCII line widths were produced using angles of *θ* as in figure 2, of 16° (*a*–*c*) and 26° (*d*–*f*), with irradiation of 6, 18 and 36 J cm^−2^. A section (cyan line) was taken across three lines of LHCII proteins in panel (*c*). (*g*) Height profile corresponding to this section.

**Figure 7.**
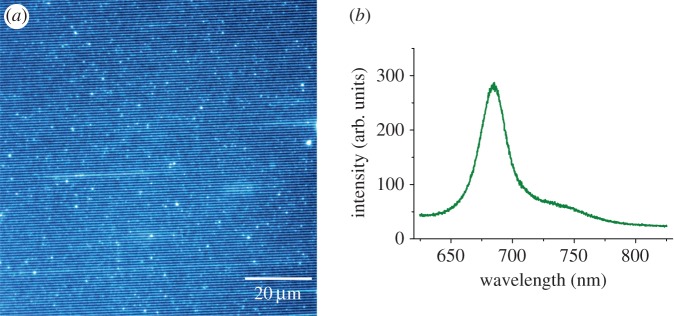
Fluorescence analyses of the LHCII complex immobilized on IL-patterned lines on gold substrates. (*a*) Fluorescence image (scale bar, 5 µm) and (*b*) the fluorescence emission spectrum.

This strategy exploits the lysine residues on the N-terminal side of the LHCII complex which, along with the use of the DMS cross-linker (see §2.2.2), ensures a consistent orientation on the amine surface. Variations in the periodic patterns were created using two different half-interference beam angles, and by altering the exposure times. The AFM topographs show the expected height of around 6–7 nm for immobilized LHCII complexes, and that linewidths of 200, 80 and 60 nm (figure 6*a*,*b*,*c*, respectively), and 200 nm, 100 nm and 60 nm (figure 6*d*,*e*,*f*, respectively), have been achieved. The background islands visible in figure 6, and also figure 4, are the ‘terraces’ or the facets of the epitaxially grown gold layer on top of the mica substrate [53,54]. The bright spots seen in the AFM images, some lying on and some between the protein nanolines, most likely result from aggregation of a small fraction of the LHCII protein. These aggregates can also be seen in the fluorescence image in figure 7*a*.

In order to verify the retained functionality of IL-patterned LHCII complexes, we imaged the fluorescent LHCII lines (figure 7*a*) and also recorded the fluorescence emission spectrum. The 470 nm excitation we used predominantly excites chlorophyll *b* and the carotenoids, whereas the fluorescence emission maximum at 684 nm is characteristic of chlorophyll *a* emission. Thus, the emission spectrum of immobilized LHCII complexes (figure 7*b*) indicates internal energy transfer from chlorophyll *b* to chlorophyll *a* and shows that the immobilized LHCII complexes retain their structural and functional integrity [43].

## 4. Conclusion

Interference lithography offers a simple, rapid and scaleable method for fabricating nanostructures of SAMs of NTA and amine-thiols on a gold substrate. By systematically varying simple processing parameters such as the energy dose and the beam interference angle, it was possible to vary the period of NTA lines from 240 to 1200 nm, and to control the width of patterned NTA lines within the range 50–600 nm. These nanostructures allow the efficient attachment of proteins; we used light-harvesting complexes from bacteria and plants because of their biological interest and their intrinsic property of binding fluorescent chlorophylls that act as sensitive reporters of their structural and functional integrity. AFM, fluorescence and spectral imaging of surface-attached light-harvesting complexes show that these proteins remain functional despite their proximity to the evanescent field of the gold substrate, which risks quenching of fluorescence. The IL patterning in this work lays the foundations for fabrication of mesoscale multi-protein assemblies for harvesting solar energy and its storage as charge-separated states at defined locations.
